# The JAK-STAT Pathway Controls *Plasmodium vivax* Load in Early Stages of *Anopheles aquasalis* Infection

**DOI:** 10.1371/journal.pntd.0001317

**Published:** 2011-11-01

**Authors:** Ana C. Bahia, Marina S. Kubota, Antonio J. Tempone, Helena R. C. Araújo, Bruno A. M. Guedes, Alessandra S. Orfanó, Wanderli P. Tadei, Claudia M. Ríos-Velásquez, Yeon S. Han, Nágila F. C. Secundino, Carolina Barillas-Mury, Paulo F. P. Pimenta, Yara M. Traub-Csekö

**Affiliations:** 1 Laboratório de Biologia Molecular de Parasitas e Vetores, Instituto Oswaldo Cruz, Fiocruz, Rio de Janeiro, Rio de Janeiro, Brazil; 2 Laboratório de Entomologia Médica, Instituto René Rachou, Fiocruz, Belo Horizonte, Minas Gerais, Brazil; 3 Laboratório de Malária e Dengue, Instituto Nacional de Pesquisas da Amazônia, Aleixo, Manaus, Amazonas, Brazil; 4 Laboratório de Biodiversidade em Saúde, Centro de Pesquisa Leônidas e Maria Deane, Fiocruz, Manaus, Amazonas, Brazil; 5 Department of Agricultural Biology, Chonnam National University, Gwangju, South Korea; 6 Laboratory of Malaria and Vector Research, National Institute of Allergy and Infectious Diseases, National Institutes of Health, Rockville, Maryland, United States of America; Colorado State University, United States of America

## Abstract

Malaria affects 300 million people worldwide every year and 450,000 in Brazil. In coastal areas of Brazil, the main malaria vector is *Anopheles aquasalis*, and *Plasmodium vivax* is responsible for the majority of malaria cases in the Americas. Insects possess a powerful immune system to combat infections. Three pathways control the insect immune response: Toll, IMD, and JAK-STAT. Here we analyze the immune role of the *A. aquasalis* JAK-STAT pathway after *P. vivax* infection. Three genes, the transcription factor Signal Transducers and Activators of Transcription (STAT), the regulatory Protein Inhibitors of Activated STAT (PIAS) and the Nitric Oxide Synthase enzyme (NOS) were characterized. Expression of STAT and PIAS was higher in males than females and in eggs and first instar larvae when compared to larvae and pupae. RNA levels for STAT and PIAS increased 24 and 36 hours (h) after *P. vivax* challenge. NOS transcription increased 36 h post infection (hpi) while this protein was already detected in some midgut epithelial cells 24 hpi. Imunocytochemistry experiments using specific antibodies showed that in non-infected insects STAT and PIAS were found mostly in the fat body, while in infected mosquitoes the proteins were found in other body tissues. The knockdown of *STAT* by RNAi increased the number of oocysts in the midgut of *A. aquasalis*. This is the first clear evidence for the involvement of a specific immune pathway in the interaction of the Brazilian malaria vector *A. aquasalis* with *P. vivax*, delineating a potential target for the future development of disease controlling strategies.

## Author Summary

Malaria is endemic in 22 countries in the Americas where the *Anopheles aquasalis* mosquito is an important malaria vector and the *Plasmodium vivax* parasite is responsible for most malaria cases. This natural vector-parasite pair is difficult to study due to the lack of cultivating system for *P. vivax*, and to the lack of genome data for *A. aquasalis*. Moreover, almost all previous studies are based on African and Asian anopheline species. Understanding the interaction mechanisms between mosquito vectors and plasmodia is important for the development of malaria control strategies. Our results showed that the JAK-STAT immune pathway is activated in *A. aquasalis* after *P. vivax* challenge and is important to maintain the low levels of *P. vivax* load observed in this vector. Our results add to the understanding of the *A. aquasalis* interaction with *P. vivax* and lead to possible explanations for this vector competence in *P. vivax* transmission. All information generated here may be used to direct the development of new or specific strategies to block malaria transmission by *A. aquasalis* in some parts of the Americas.

## Introduction

Malaria is one of the most important vector-borne diseases, affecting 300 million people worldwide every year and 22 countries in America. Brazil presents over half of the total estimated cases with numbers varying from 300 to 600 thousand over the past years [1]. The lack of effective vaccines, the development of drug resistance in *Plasmodium* parasites and of insecticide resistance in mosquitoes, have prevented the successful control of human malaria in many tropical regions. Understanding the biology of the *Plasmodium*-mosquito vector interaction is important to identify potential targets for the development of novel malaria control strategies to disrupt the parasite life cycle in the insect vectors and prevent disease transmission to humans. The mosquito immune system limits parasite development and over-activation of some immune pathways has been shown to decrease *Plasmodium* infection [2], [3].

The insect immune system is very efficient in defending against a diversity of pathogens through multiple innate immune responses, which are also present in higher organisms [4]. Genetic studies in *Drosophila* identified three major signaling pathways that regulate expression of immune effector genes: TOLL, Immune deficiency (IMD), Janus Kinase and Signal Transducer and Activator of Transcription (JAK-STAT) pathways [5]. In mosquitoes it was demonstrated that the Imd pathway prevents the development of *Plasmodium falciparum* in *Anopheles gambiae*, *Anopheles stephensi* and *Anopheles albimanus* while the Toll pathway is most efficient in *A. gambiae* against *Plasmodium berghei* [2], [3].

The JAK-STAT pathway was first described as a cytokine induced intracellular signaling pathway [6], [7] very tightly regulated by a series of activators and suppressors. In humans, over-activation of this pathway has been associated with neoplastic transformation [8]. In *Drosophila*, the JAK-STAT pathway has been implicated in several cellular processes such as regeneration, homeostasis, eye development and embryonic segmentation. In addition, in *Drosophila* this pathway participates in some cellular immune responses as differentiation of prohemocytes and hemocyte proliferation, as well as in antibacterial responses [9]–[12]. Recent studies showed that the JAK-STAT pathway mediates *Anopheles gambiae* immune response to *P. berghei* and *P. falciparum* [13] and *Aedes aegypti* response to dengue virus II [14].

In *Drosophila melanogaster*, activation of the STAT pathway is initiated when the peptide ligand Unpaired (Upd) binds to the transmembrane receptor Domeless. This activates the JAK kinase Hopscotch to phosphorylate the transcription factor Stat92E. The phosphorylated STAT protein forms a dimer, translocates to the nucleus and activates transcription of target genes [10]. This pathway is tightly regulated by various proteins, such as Suppressor of Cytokine Signaling (SOCS) and Protein Inhibitor of Activated STAT (PIAS). The SOCS gene is transcriptionally activated by the STAT pathway as part of a negative feedback loop that modulates STAT signaling by preventing STAT phosphorylation, while PIAS inhibits signaling by directly binding to STAT proteins and targeting them for degradation [15].

*Anopheles aquasalis* is an important malaria vector in the Brazilian coast. Although *Plasmodium vivax* is more widely distributed than *P. falciparum*, and there are close to three billion people at risk of infection by this parasite worldwide [16], research on the biology and transmission of *P. vivax* has been neglected for several decades. This is mostly due to the lack of an efficient continuous cultivation system and to the misconception that this parasite does not cause severe malaria [17], [18]. Although it has long been considered a benign infection, it is now accepted that *P. vivax* can cause severe and even lethal malaria [19].

We cloned and characterized three genes from the JAK-STAT pathway: the transcription factor *STAT*, the *PIAS* regulatory proteins and the enzyme *NOS*. The main goal of this study was to determine whether the JAK-STAT pathway is activated in *A. aquasalis* in response to *P. vivax* infection and, if so, whether this response limits *Plasmodium* infection.

## Methods

### Ethics statement

For the acquisition of *P. vivax* infected human blood, patients were selected among people visiting the Health Center (Posto Estadual de Saúde da Vigilância em Saúde do Município de Iranduba, Distrito de Cacau Pirêra, Amazonas, Brazil) searching for malaria diagnosis and treatment during outbreaks. Diagnosis was performed by Giemsa stained blood smears. After *P. vivax* positive diagnosis with presence of about 4–8% circulating gametocytes, patients were interviewed and inquired about the possibility of volunteer donation of a small amount of blood for research purposes. Subsequently, a patient consent form was first read to the potential volunteers, with detailed verbal explanation, and signed by all patients involved in the study. After this agreement, 200 microliters of venous blood was drawn from each patient and placed in heparinized tubes. Blood samples were kept under refrigeration in an icebox (at approximately 15°C) for about 15 minutes, taken to the laboratory and used to feed *A. aquasalis*. All ethical issues of this study followed international rules including the Declaration of Helsinki. The used protocols, including the human consent forms, were previously approved by the Brazilian Ministry of Health, National Council of Health, National Committee of Ethics in Research (CONEP) (written approval number 3726).

### Insect infection

*A. aquasalis* were reared at 27°C and 80% humidity [20]. Insect infections were performed in a safety insectary at an endemic area of Manaus, Amazonas state, as described in Bahia *et al.* [21]. Human infected blood containing 4–8% gametocytes or normal blood (control) were offered to the insects by oral feeding using a membrane glass feeder device under constant 37°C temperature, maintained using a water circulation system, to prevent exflagellation of microgametocytes [21]. After the experimental feeding, mosquitoes were kept in cages at 27°C and given 20% sucrose *ad libitum*. Mosquito infection was evaluated by PCR using a specific *Plasmodium* 18s rRNA gene [21].

The experimental prevalence rate of infected *A. aquasalis* mosquitoes with *P. vivax* was 36%, as detected by PCR or by oocysts presence (number of infected mosquitoes/total examined). The mean intensity of the infected mosquitoes was 7.6% (i.e., the average number of parasites as calculated using the number of infected mosquitoes as the denominator). A low number of *P. vivax* oocysts were consistently found in the infected mosquitoes, which is in agreement with the usual low number of human malaria parasites found infecting mosquito vectors in nature.

### PCR using degenerate primers

PCR reactions were performed as described using degenerate primers designed on conserved regions of *STAT* and *PIAS*, based in sequences of *A. gambiae*, *A. stephensi*, *A. aegypti* and *D. melanogaster* [22]. The PCR cycles used were: two cycles (1 min steps at 95°C, 55°C and 72°C, and 95°C, 42°C and 72°C) followed by 30 cycles at moderate stringency (1 min steps at 95°C, 52°C and 72°C) and a final 7 min extension at 72°C. All amplicon generated were cloned into pGEM®-T Easy Vector (Promega) and utilized to transform high efficiency DH5-α *Escherichia coli*. Sequencing of the selected clones was performed using an ABI 3700 sequencer (Applied Biosystems) and the ABI PRISM® BigDye™ Terminator Cycle Sequencing reagent (Applied Biosystems) in the PDTIS/FIOCRUZ Sequencing Platform.

### RACE

The SMART cDNA RACE amplification kit (Becton Dickinson Clontech) was used to obtain the 5′ and 3′ ends of the PIAS and STAT cDNAs. All amplicons generated were cloned and sequenced as described above. After sequencing, the cDNAs of STAT and PIAS were assembled using the CAP3 Sequence Assembly Program (http://pbil.univ-lyon1.fr/cap3.php) and aligned with other insect sequences with the Clustal W Program (http://www.ebi.ac.uk/Tools/clustalw2/).

### Real Time PCR (RTPCR)

RNA was extracted from whole insects submitted to different experimental conditions (immature stages – egg, first to fourth instar larvae and pupa; sugar-fed males and females; females fed on blood and blood from *P. vivax* malaria patients). The extracted RNA was treated with RQ1 RNAse-free DNAse (Promega) and utilized for cDNA synthesis. RTPCR reactions were performed using the SyberGreen fluorescent probe employing an ABI 7000 machine (Applied Biosystems). The PCR cycles used were 50°C 2 min, 95°C 10 min, 95°C 15 sec and 63°C 1 min for 35 times for all reactions. The primer sequences were: STATFwd 5′ CTGGCGGAGGCGTTGAGTATGAAAT 3′ and STATRev 5′ CGGATAAGGAAGGCTCGTTTTGAAT 3′, PIASFwd 5′ TAGCAGCTCACAGTATAGCCTCGAT 3′ and PIASRev 5′ TCCCATTCCAACCAACAAACCA 3′, and NOSFwd 5′ AGGATCTGGCCCTCAAGGAAGCCGA 3′ and NOSRev 5′ ATCGTCACATCGCCGCACACGTACA 3′. The relative expression of the selected genes was based on gene expression CT difference formula [23]. Quantifications were normalized in relation to the housekeeping gene rp49 [24]. All the experiments were performed using four to six biological replicates and three experimental replicates. The Shapiro-Wilk and Levene tests were used to determine when parametric versus non-parametric tests should be used. The ANOVA test with multiple comparisons of Tukey or Games-Howell was used in the analyses. When this parametric model was not adequate, the Kruskal-Wallis test with multiple comparisons of Dunn's was utilized. Bonferroni correction was used when necessary. All tests were performed with reliable level of 95% (α = 0.05). The statistical analyses were accomplished using the GraphPad Prism5® and R 2.9.0.

### Western blot

Proteins of whole insects submitted to different feeding regimens (sugar-fed males and females, and females after different times of blood feeding and infection) were extracted by Trizol Reagent (Invitrogen) following the manufacturer's “instructions for protein isolation” protocol. Samples corresponding to one insect were separated on 12% SDS-PAGE gels and subsequently transferred to Hybond nitrocellulose membranes. The membranes were blocked with 5% non-fat milk TBS Tween 20 0.1% (TBST) for at least one hour. The membranes were then incubated with anti-PIAS antibody at a 1∶250 dilution for two hours. After three washes of 10 minutes in TBST, the membranes were incubated with anti-rabbit secondary antibody at a 1∶80.000 dilution for one hour. Three more washes were performed before the incubation of the membrane with the detection system Pierce SuperSignal West Pico chemiluminescent substrate (ThermoScientific).

### Immunocytochemistry

Sugar-fed male and female *A. aquasalis* submitted to different treatments (sugar-feeding, infected and non-infected blood-feeding) were collected, had their heads, legs and wings removed, and were fixed overnight at 25°C in 4% paraformaldehyde in PBS. The insects were dehydrated in 30% to 100% ethanol, and then infiltrated with Hystoresin kit (Leica) at room temperature for 5–7 days. Hystoresin-embedded mosquitoes were transversally sectioned using a rotary microtome in order to expose the organs located in the abdomen and thoracic regions. The 3 µm-thick sections were adhered to slides, dried, incubated for 20 minutes in 1% PBS/BSA and 20 minutes in RPMI medium in order to avoid nonspecific antibody binding. Sections were then incubated overnight with 1∶250 anti-rabbit STAT or PIAS antibodies diluted in 1% PBS/BSA. The tissue sections were washed 5–8 times with 1% PBS/BSA and then incubated with rabbit secondary antibody conjugated to FITC (Molecular Probes), diluted 1∶250 in blocking solution. The same steps were performed in the control samples, except for the incubation with the primary antibody After two washes in PBS, the slides were mounted using Mowiol anti-photobleaching Mounting Media (Sigma Aldrich). Immunostaining was analyzed with a confocal laser microscope (Zeiss-LMS 510). Photos are representative of at least five mosquitoes for each treatment. Alternatively, midguts of females 24 hpi were dissected, opened transversely in order to expose the lumen and fixed for 20 minutes (m) in 4% paraformaldehyde in PBS at 4°C in order to be processed for immunocytochemistry as described elsewhere [25]. The opened insect midguts were treated with 1% PBS/BSA followed by RPMI medium as described above. Then, the tissue sections were incubated with commercial anti-NOS antibody (Sigma Aldrich SAB4300426) diluted 1∶250 in 1% BSA/PBS. Five washes were performed and the midguts were incubated with anti-rabbit antibody conjugated to Alexa 594 diluted 1∶250 in 1% PBS/BSA. Five more washes with PBS were performed before mounting the midguts in slides with Mowiol. The same steps were performed in the control samples, except for the incubation with the primary antibody. The material was analyzed by confocal laser microscopy.

### Gene silencing

Double stranded RNAs (dsRNAs) for *STAT* (dsSTAT) and ß*-ga*l (dsβ-gal) were produced from PCR-amplified fragments using the T7 Megascript kit (Ambion). Amplicons for dsß-gal were produced using plasmid templates and for dsSTAT by reverse transcriptase PCR (RT-PCR) products, from sugar-fed female cDNA, giving rise to 544 bp and 503 bp fragments, respectively. Two rounds of PCR were performed to amplify ß-gal. The first PCR round was performed with primers containing a short adaptor sequence at the 5′ end (tggcgcccctagatg). The primers used for the first round of PCR were ß-galFwd 5′tggcgcccctagatgTGATGGCACCCTGATTGA 3′ and ß-galRev 5′ tggcgcccctagatgTCATTGCCCAGAGACCAGA 3′. The PCR cycles utilized were 95°C for 3 min, 35 cycles of 95°C for 30 s, 57°C for 45 s and 72°C for 45 s followed by 72°C for 7 min. Two microliters of the first PCR were used in the second PCR reaction. The second round of PCR was utilized to insert the bacteriophage T7 DNA-dependent RNA polymerase promoter to the DNA templates. The second round of PCR utilized the same conditions of the first reaction. The second round PCR primer, which has the T7 (bold letters) and the adaptador sequences, was 5′ ccg**TAATACGACTCACTATAGG**tggcgcccctagatg 3′. STAT amplification was performed in one round of PCR, which also inserted the T7 sequence. The STAT primer used was STATFwd 5′ **TAATACGACTCACTATAGG**GGATGATGTACCGGACCTGCT 3′ and STATRev 5′ **TAATACGACTCACTATAGG**GGTGTACGATGACGACAACCG 3′. The amplification of the STAT sequence was done using the PCR cycles as follows: 95°C for 5 min and 35 cycles of 95°C for 30 s, 55°C for 45 s and 72°C for 45 s.

dsSTAT or dsß-gal (69 nL of 3 µg/µL) diluted in water were introduced into the thorax of cold anesthetized 3–4 day old female mosquitoes by a nano-injector (Nanoject, Drummond) with glass capillary needles. After the injection, the insects were maintained in an air incubator and fed on sugar solution.

At two to three days after the dsRNA injections, the insects were fed with *P. vivax* infected blood. Three to five days after infection, the oocysts in the basal lamina of the gut epithelium were counted to estimate the *P. vivax* load in the infected mosquito. Each dissected mosquito gut was stained with 2% mercurochrome and observed under light microscopy. At least 30 guts were used for each experimental condition and three different gene silencing experiments were performed. Oocyst numbers in dsSTAT injected insects were compared to insects injected with β-gal dsRNA, a control for a gene not found in the insect. The significance of gene silencing effect on oocysts loads was determined by the Mann-Whitney statistical test.

### Semi-quantitative RT-PCR

Total RNA was extracted from females, either sugar-fed or one to five days after dsRNA injections. Up to 5 µg of RNA were treated with RQ1 RNAse-free DNAse (Promega) and used for first strand cDNA synthesis utilizing the ImProm-II™ Reverse Transcription System (Promega). PCR reaction conditions were the same utilized for RTPCR, as were the primers (STAT and rp49). Biological and experimental triplicates were performed. The PCR reactions were separated in a 2.5% ethidium bromide-containing agarose gel. The housekeeping gene rp49 was used to normalize the reactions [24] and sugar-fed female samples were used as reference samples. The intensity of amplified products was measured using ImageJ 1.34 s software (http://rsb.info.nih.gov/ij) and plotted for semi-quantitative analysis. The ANOVA test was used as statistics method.

## Results

### Identification and characterization of *A. aquasalis* STAT and PIAS

Two genes of the JAK-STAT pathway of *A. aquasalis*, the transcription factor *STAT* (AqSTAT) and its regulatory protein *PIAS* (AqPIAS) were amplified by PCR, using degenerate primers and genomic DNA as template. The 1150 bp (STAT) and 891 bp (PIAS) PCR fragments were cloned and sequenced. After *in silico* predictions of exons and introns, 836 bp and 549 bp coding sequences were obtained for STAT and PIAS, respectively. These sequences were used to design perfect-matching primers and the SMART RACE technique was used to obtain the complete cDNA sequences of these two genes using a mixture of cDNAs from males and infected and non-infected females as template. A full-length AqSTAT cDNA sequence of 1599 bp was obtained, consisting of a 1491 bp open reading frame (ORF) coding for a 497 amino acid residues protein, plus a 108 bp 3′ untranslated region (UTR) (Figure S1). The full-length AqPIAS cDNA consists of 2407 bp including a 1953 bp ORF, which encodes a protein of 651 amino acid residues, as well as a 211 bp 5′ UTR and 243 bp 3′ UTR (Figure S2). These two sequences were deposited in GenBank with accession numbers HM851178 and HM851177, respectively.

**Figure 1:**
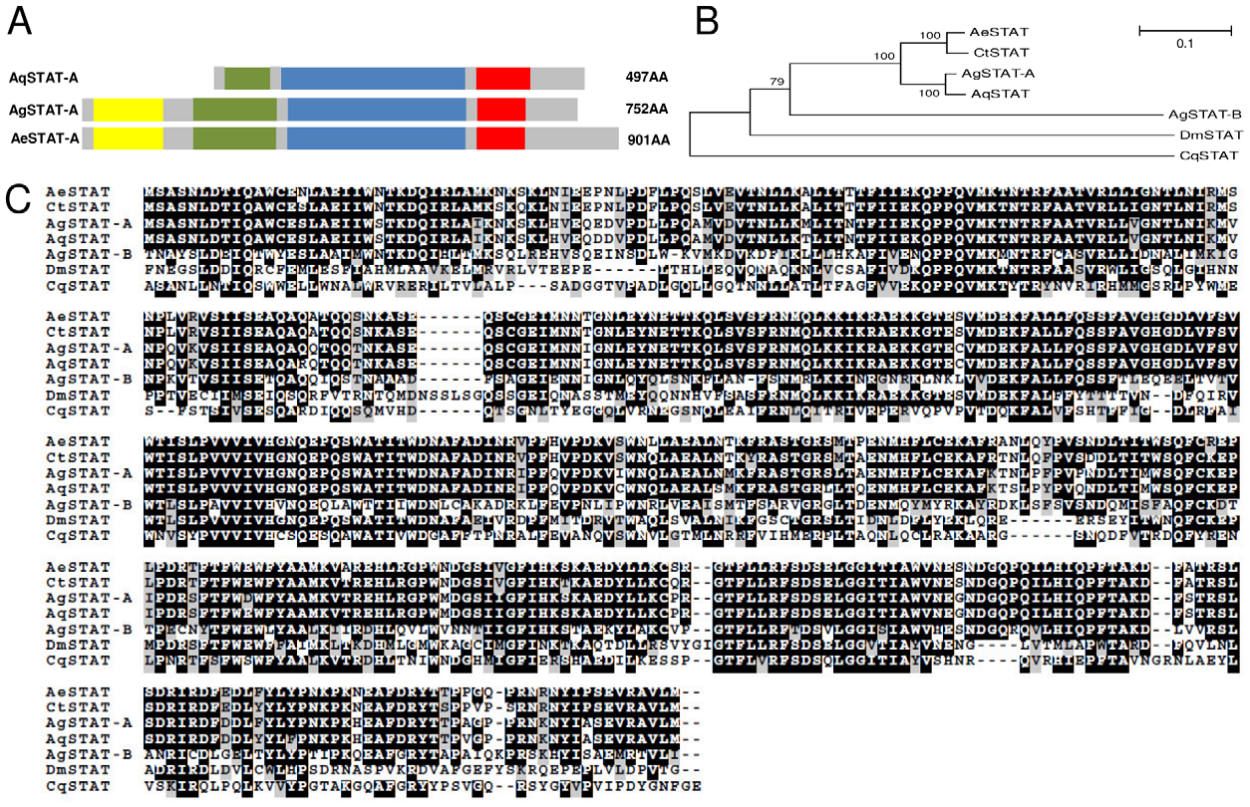
Characterization of the *STAT* gene. A: Schematic representation of STAT protein from *A. aquasalis* (AqSTAT-A), *A. gambiae* (AgSTAT-A and AgSTAT-B) and *A. aegypti* (AeSTAT-A) showing the STAT interaction domain (yellow), STAT alpha domain (green), STAT binding domain (blue) and SH2 domain (red). B: Phylogenetic tree for STAT using insect sequences, constructed based on the neighbor-joining method. C: Multiple aminoacid sequence alignment of STAT of insects. Accession numbers of STAT sequences from: *A. aquasalis* (Aq) – HM851178, *A. gambiae* (Ag) (STAT-A – ACO05014.1 and STAT-B – CAA09070.1), *A. aegypti* (Ae) – ABO72629.1, *Culex quinquefasciatus* (Cq) – XP_001866606.1, *Culex tritaeniorhynchus* (Ct) – AAQU64663.1, and *D. melanogaster* (Dm) – NP_996243.1.

**Figure 2:**
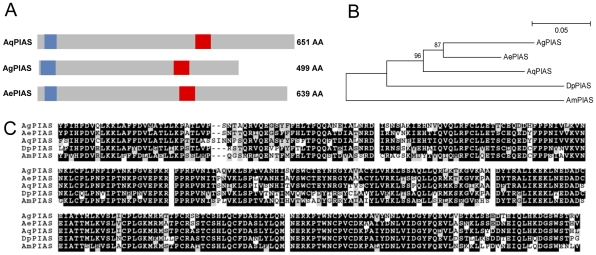
Characterization of *PIAS* gene. A: Schematic representation of *A. aquasalis* (Aq), *A. gambiae* (Ag) and *A. aegypti* (Ae) PIAS proteins showing the SAP domain (blue) and the MIZ/SP-RING zinc finger domain (red). B: Phylogenetic tree for *PIAS* of insects constructed based on the neighbor-joining method. C: Multiple aminoacid sequence alignment of *PIAS* from insects. Accession numbers of PIAS sequences from: *A. aquasalis* (Aq) – HM851177, A. *gambiae* (Ag) – XP_001688469.1, *A. aegypti* (Ae) – XP_001647815.1, *D. pseudobscura* (Dp) – XP_002138569, and *A. mellifera* (Am) – XP_623571.

Sequence analyses and comparison with other mosquito STATs showed that AqSTAT presents the SH2 domain, the STAT binding domain and a portion of the alpha domain, but lacks the STAT interaction domain, presented schematically in Figure 1A. Phylogenetic approaches showed that AqSTAT grouped with STATs from other mosquitoes and was more closely related to *A. gambiae* STAT-A (the ancestral gene) than to STAT-B (a gene duplication that probably resulted from a retro-transposition even) (Figure 1B and C). AqPIAS presents two very conserved domains, the SAP domain and the MIZ/SP-RING zinc finger domain (Figure 2A). The deduced AqPIAS protein had higher homology to putative ortholog genes from other mosquitoes than to those of other insects, such as *Drosophila pseudoobscura* and *Apis mellifera* (Figure 2B and C).

**Figure 3:**
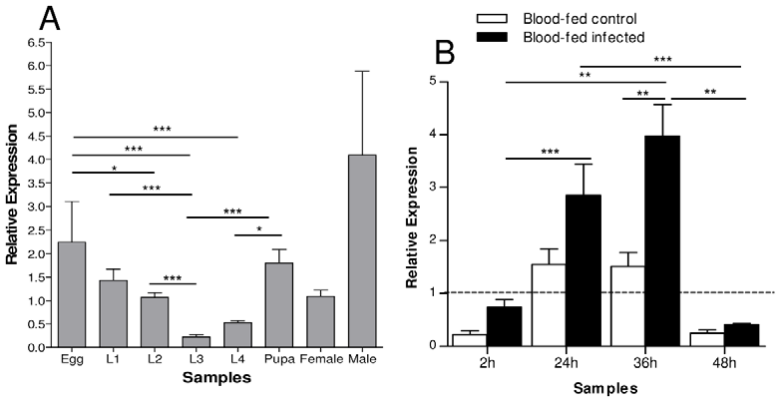
Transcription levels of *A. aquasalis* STAT determined by RTPCR. A: immature stages (eggs, larvae (L1–L4) and pupae), sugar-fed males (♂) and females (♀), B: sugar-fed females (dotted line), blood-fed control (BFC) and blood-fed infected females (BFI). h – hours, L1 – first instar larva, L2 – second instar larva, L3 – third instar larva and L4 – fourth instar larva. +–: s.e.m.; * 0.05>p>0.03, ** 0.03>p>0.01, *** p>0.01. The ANOVA test with multiple comparisons of Tukey or Games-Howell was used in A. In B the ANOVA test with multiple comparisons of Tukey or Games-Howell was used in the comparisons between the blood-fed samples analyses and the Kruskal-Wallis test with multiple comparisons of Dunn's in the blood-infected samples analyses. Bonferroni correction was used when necessary in the analyses of the blood-infected samples.

**Figure 4:**
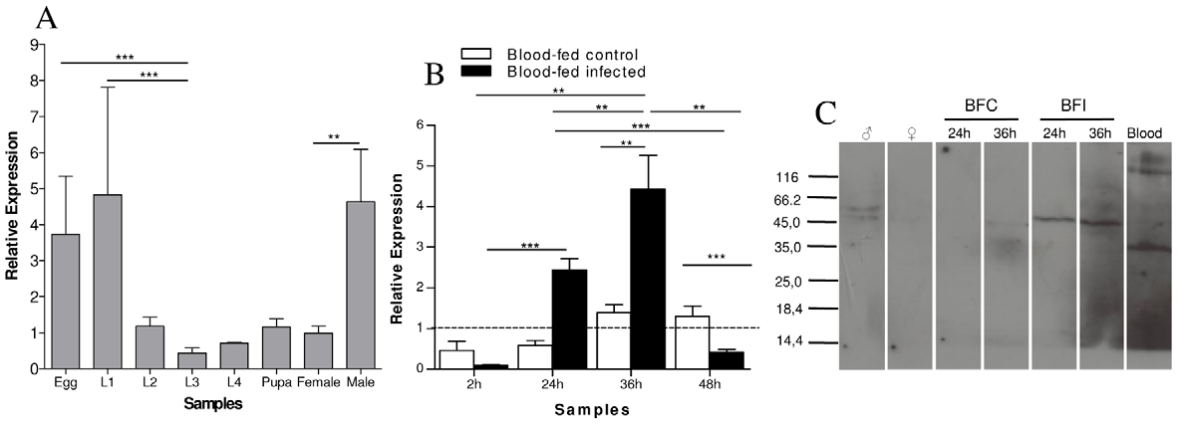
Expression of PIAS in *A. aquasalis*. A: Transcription levels of *A. aquasalis* PIAS in immature stages (eggs, larvae (L1–L4) and pupae), sugar-fed males and females, B: Transcription levels of *A. aquasalis* PIAS in sugar-fed females (dotted line), and females after blood-feeding and after *P. vivax* infection, C: Expression of PIAS protein by western blot in *A. aquasalis* submitted to different feeding regimens (sugar-fed male (♂) and female (♀), blood-fed (control) (BFC) and blood-fed infected (BFI) females) and human blood. h – hours, L1 – first instar larva, L2 – second instar larva, L3 – third instar larva and L4 – fourth instar larva. +–: s.e.m.; * 0.05>p>0.03, ** 0.03>p>0.01, *** p>0.01. The ANOVA test with multiple comparisons of Tukey or Games-Howell was used in A. In B, the ANOVA test with multiple comparisons of Tukey or Games-Howell was used in the comparisons between the blood-fed samples analyses and the Kruskal-Wallis test with multiple comparisons of Dunn's in the blood-infected samples analyses. Bonferroni correction was used when necessary in the analyses of the blood-infected samples.

Gene expression of AqSTAT and AqPIAS investigated by RTPCR revealed that these genes are expressed in all mosquito developmental stages, including adults of both genders. Transcript levels of STAT are high in eggs (Figure 3A) while PIAS has high transcription levels in both eggs and first instar larvae (Figure 4A). In adult stages, both STAT and PIAS were transcribed at higher levels in males than in females (Figures 3A and 4A). We investigated the effect of *P. vivax* infection on expression of these two genes. To circumvent the inability to culture *P. vivax*, all mosquitoes used in these studies were fed on blood drawn from human donors infected with *P. vivax* malaria. Both STAT and PIAS genes were transcriptionally activated by *P. vivax* infection at 24 and 36 hours post-infection (hpi). This induction was transient and was no longer observed 48 hpi (Figures 3B and 4B). Furthermore, PIAS protein expression was also higher in protein homogenates obtained from infected females 24 and 36 hpi (Figure 4C).

### Identification and characterization of the effector gene nitric oxide synthase

**Figure 5:**
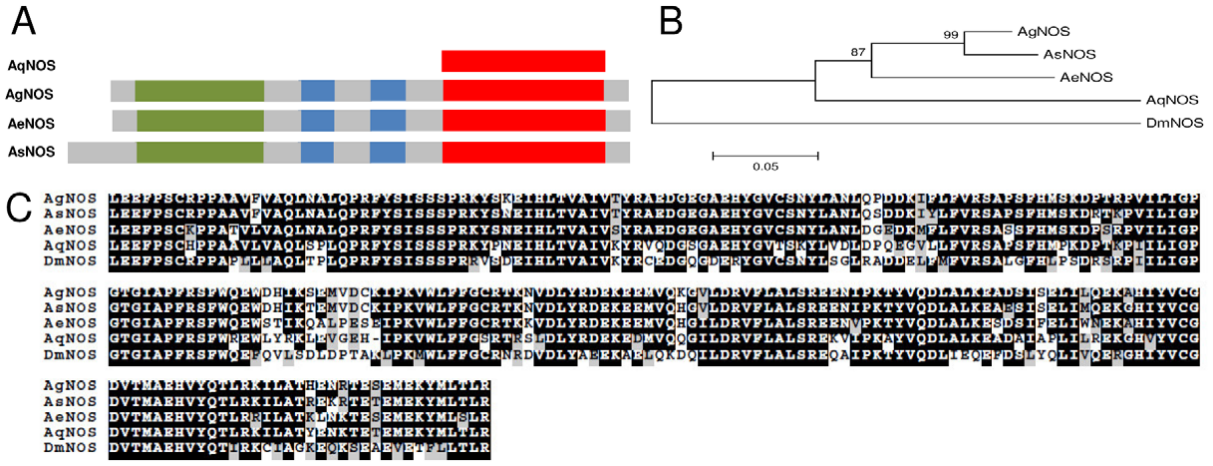
Characterization of *NOS* gene. A: Schematic representation of *A. aquasalis* NOS protein showing nitric oxide synthase (green), NADPH-dependent FMN reductase (red) and ferrodoxin reductase (red) domains. B: Phylogenetic tree of insects NOS constructed based on the neighbor-joining method. C: Multiple aminoacid sequence alignment of insects NOS. Accession numbers of NOS sequences from: *A. aquasalis* (Aq) – HM851179, *A. gambiae* (Ag) – AGAP008255-PA, *A. aegypti* (Ae) – AAEL009745, *A. stephensi* (As) – O61608, and *D. melanogaster* (Dm) – CG6713.

**Figure 6:**
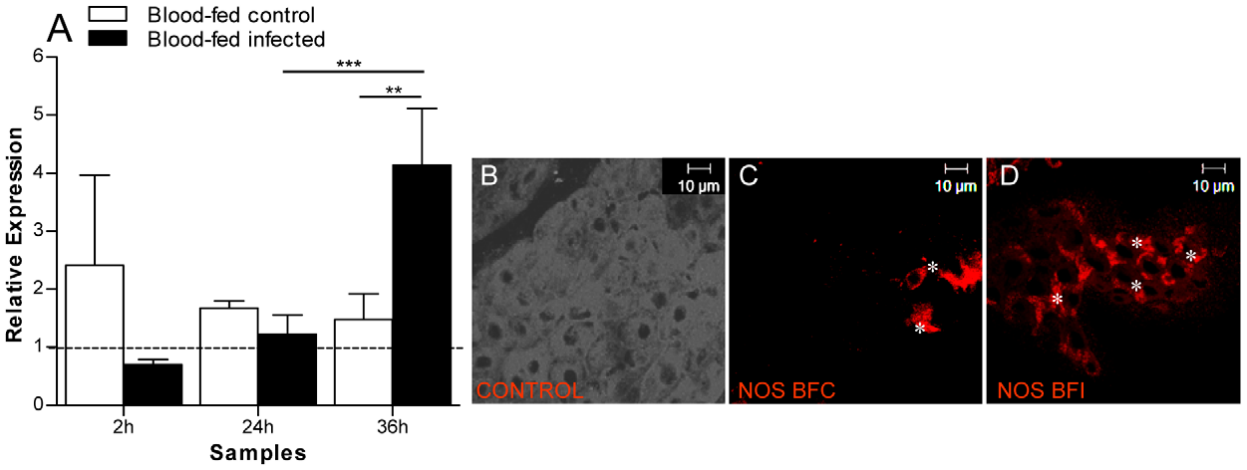
Expression of NOS in *A. aquasalis.* A: Transcription of NOS in *A. aquasalis* following different feeding regimens determined by RTPCR. A: sugar-fed females (dotted line), blood-fed control (BFC) and blood-fed infected (BFI) females. h – hours. *0.05>p>0.03, ** 0.03>p>0.01, *** p>0.01. The ANOVA test with multiple comparisons of Tukey was used in the analyses. B: Light microscopy of a transversally open midgut of *A. aquasalis* showing the gut epithelium composed by a single cell monolayer. C and D: Immunofluorescence staining of 24 hours BFC and BFI female guts with a universal anti-NOS antibody showing fluorescent epithelial cells (asterisks) positive for the presence of NOS protein.

A 702 bp cDNA fragment of *A. aquasalis* NOS (AqNOS) was obtained using degenerate primers, cloned and sequenced. This fragment is part of the nitric oxide synthase domain of NOS proteins (Figure 5A). The *A. aquasalis* NOS is closely related to mosquitoes' NOS (Figure 5B and C). This sequence was deposited in GenBank with accession number HM851179. NOS mRNA expression was induced by *P. vivax* infection 36 hpi (Figure 6A). Immunocytochemistry of *A. aquasalis* midguts infected with *P. vivax* 24 hpi revealed high levels of NOS protein expression in the cytoplasm of some epithelial cells when compared to the blood- fed insects (Figure 6B and C).

### Immunocytochemistry location of STAT and PIAS

**Figure 7:**
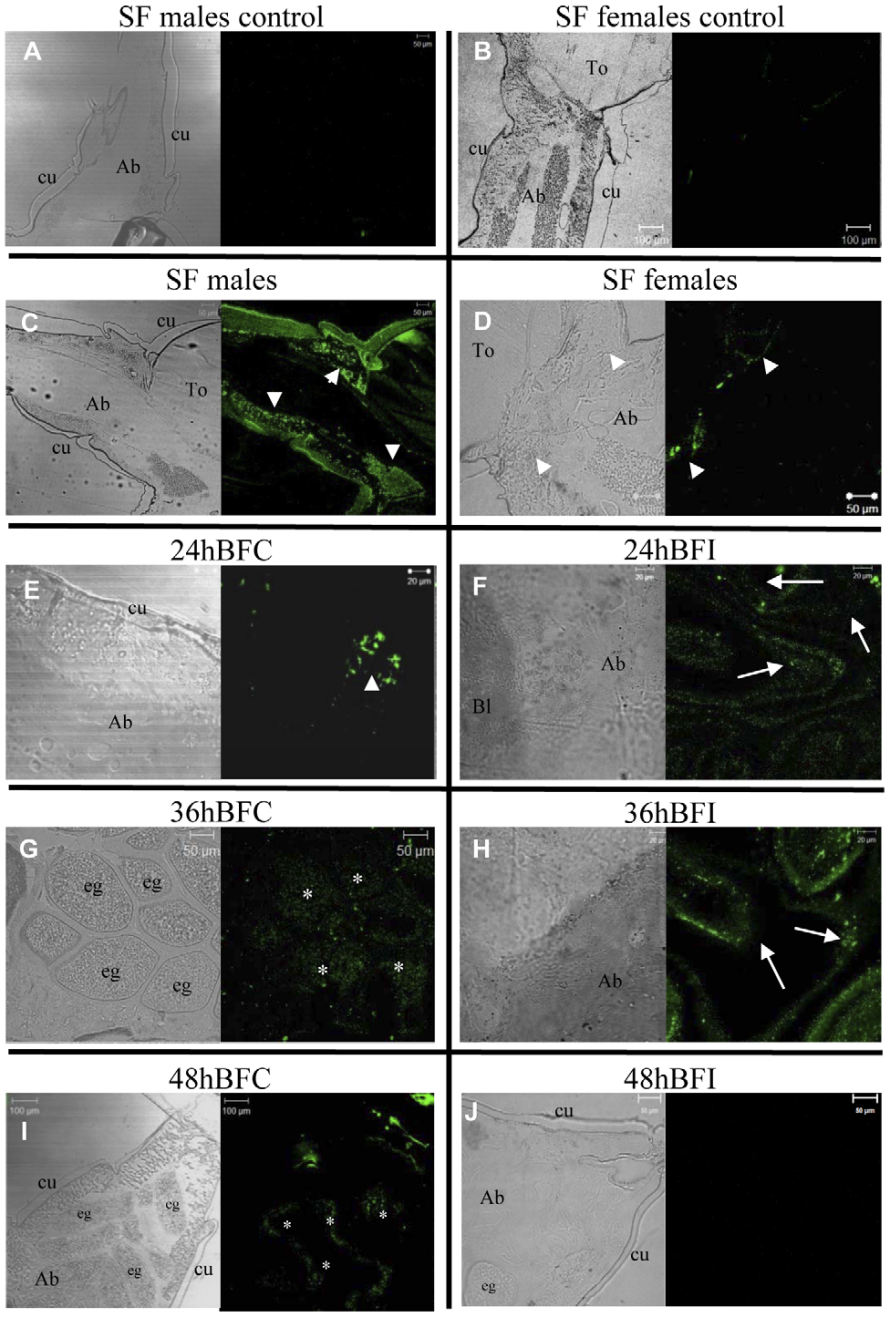
Detection of STAT protein in different tissues of *A. aquasalis*. A, B, C and D: the figures show the expression of the STAT proteins in sugar-fed (SF) males and females. A and B - control figures. E–J: the figures show the expression of the STAT proteins in females submitted to different feeding regimes. E, G and I – 24, 36 and 48 hours (h) blood-fed control (BFC), respectively; G F, H and I - 24, 36 and 48 h blood-fed infected (BFI), respectively. Arrowheads show the fat body, asterisks represent the eggs and arrows represent disperse cells expressing STAT proteins. To - thorax, Ab - abdomen, eg - eggs and Bl - blood.

**Figure 8:**
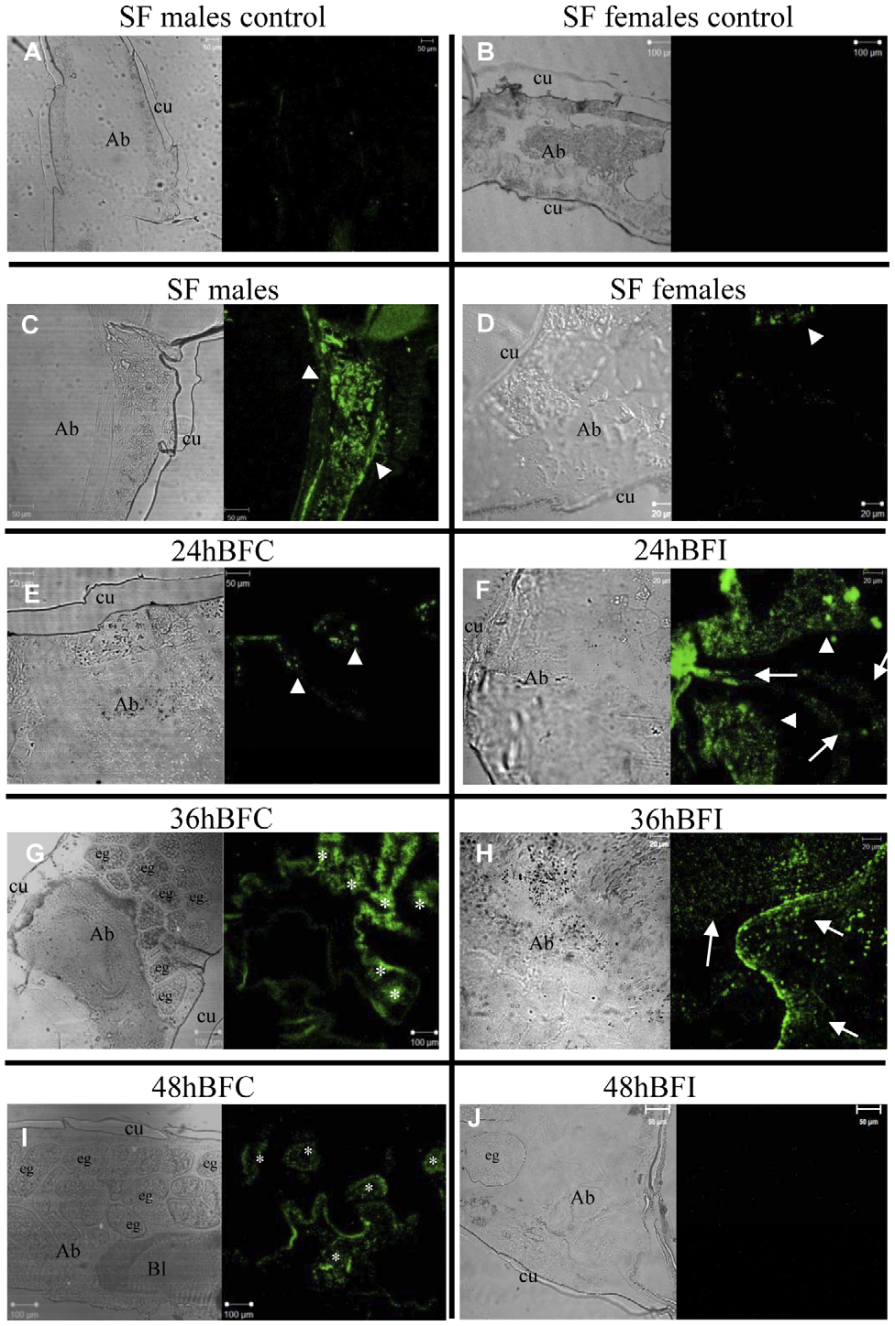
Expression of PIAS in different tissues of *A. aquasalis* insects. A, B, C and D: the figures show the expression of the PIAS proteins in sugar-fed (SF) males and females. A and B - control figures. E–J: the figures show the expression of the PIAS proteins in females submitted to different feeding regimes. E, G and I – 24, 36 and 48 hours (h) blood-fed control (BFC), respectively; G F, H and I - 24, 36 and 48 h blood-fed infected (BFI), respectively. Arrowheads show the fat body, asterisks represent the eggs and arrows represent disperse cells expressing PIAS proteins. To - thorax, Ab - abdomen, eg - eggs and Bl - blood.

To reveal the tissues responsible for the expression of STAT and PIAS proteins, immunocytochemistry experiments were carried out. Antibodies against STAT and PIAS labeled distinctly the tissue sections of *A. aquasalis* according to the experimental conditions. There was very little nonspecific labeling in tissue sections of male (Figures 7A and 8A) or female (Figures 7B and 8B) control mosquitoes submitted only to incubation with secondary fluorescent antibodies. In sugar-fed mosquitoes, while males presented STAT and PIAS immunolabeling in several body parts, noticeably in the fat body (Figures 7C and 8C), both proteins expression was weaker in sugar-fed females (Figures 7D and 8D). In non-infected blood-fed females at 24 h, 36 h and 48 h, immunolabeling for both STAT (Figure 7E, 7G and 7I) and PIAS (Figure 8E, 8G and 8I) was mainly in the fat body and eggs. The labeling intensity increased with time, with fluorescence peak at 36 h (Figure 7G and 8G), remaining noticeable at 48 h (Figures 7I and 8I), the last time point used in our experiments. However, in *P. vivax* infected mosquitoes, immunolabeling of both STAT and PIAS appears to be stronger than the non-infected mosquitoes at 24 h (Figures 7F and 8F) and 36 h (Figures 7H and 8H), but no detectable fluorescence was seen at 48 h (Figures 7J and 8J). This corroborated our mRNA and protein expression results.

### Silencing of STAT

**Figure 9:**
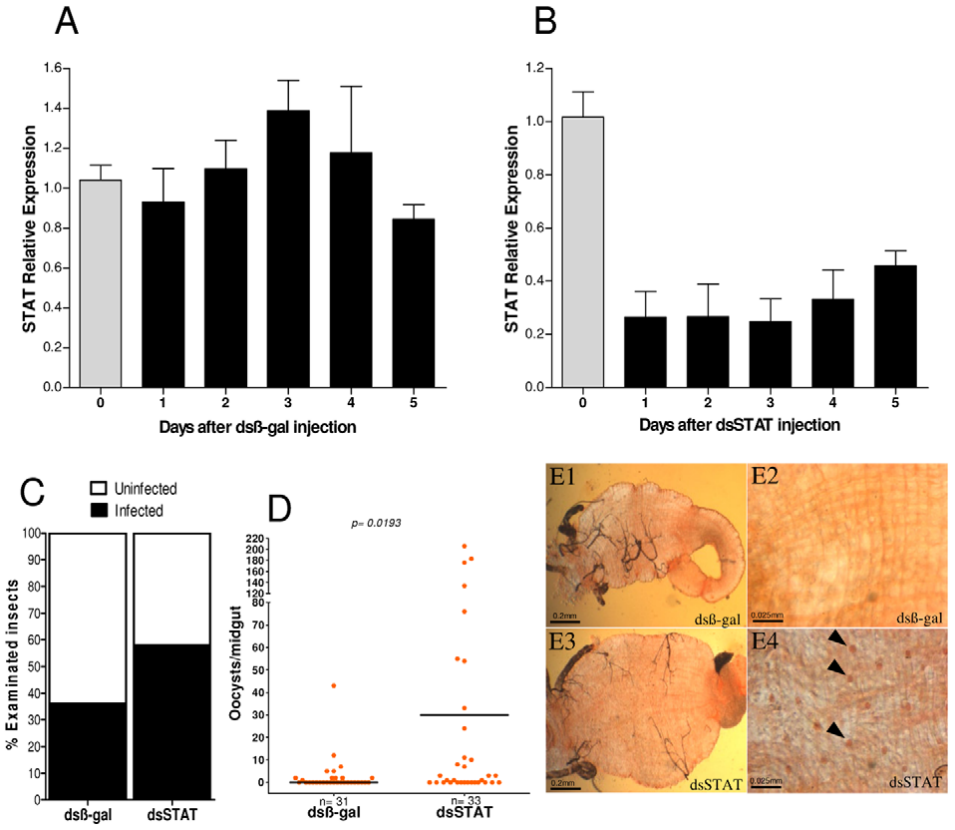
Effect of STAT silencing on *A. aquasalis* susceptibility to *P. vivax* infection. A and B - Effect of dsRNA-mediated knockdown of *ß-gal* (control) and *STAT* on *A. aquasalis* STAT expression 1 to 5 days after dsRNA injection evaluated by semi-quantitative PCR. Day zero refers to *A. aquasalis* sugar-fed females. C- Number of infected insects after dsRNA injections. D and E - Oocyst numbers (D) and visualization (arrows) (E) in midguts of mosquitoes previously injected with dsRNAfor ß-gal (D, E1 and E2) and STAT (D, E3 and E4) three to five days after *Plasmodium* infection. Three replicates of each experiment were performed. The significance of gene silencing on oocyst load in experimental samples, compared to dsß-gal-treated controls, was determined by Mann-Whitney statistical test with Bonferroni correction.

To test whether activation of the JAK-STAT pathway limits *P. vivax* infection in *A. aquasalis*, the effect of silencing the transcription factor AqSTAT by systemic injection of dsRNA was evaluated. As a control, females were injected with dsß-gal, a gene not present in the mosquito genome. The transcription level of STAT was greatly reduced (70%) in mosquitoes injected with dsSTAT, relative to those injected with dsß-gal (Figure 9A and B). This effect was already observed one day post-injection and was still present 5 days post-injection. Mosquitoes were infected with *P. vivax* two to three days after dsRNA injection. Three to five days after infection, the guts were dissected and the oocysts were counted. These experiments revealed that reducing expression of the STAT gene increased the proportion of infected *A. aquasalis* females as well as oocysts density (Figure 9C, D and E).

## Discussion

The JAK-STAT pathway is very conserved among species all the way from insects to humans. This pathway is important in insect immune response against some pathogens as bacteria [26]–[28], virus [14], [29] and *Plasmodium* [13]. A single STAT gene (STAT92E) was found in *Drosophila* as well as several other components of this signaling pathway such as: two homologous receptor ligands (*Upd2* and *Upd3*), a membrane receptor (*Domeless*) and a JAK-kinase homologue (*Hopscotch*) [10]. Some JAK-STAT repressors have also been characterized in *D. melanogaster*, as for example SOCS (*SOCS36E*) [30] and PIAS (*dPIAS*) [31]. Bioinformatics analysis of the *A. aegypti* and *A. gambiae* genome sequences revealed the existence of Domeless, Hopscotch, STAT, PIAS and SOCS orthologs in these two mosquito species [14], [31]. All dipteran insects examined so far have a single STAT gene, except for *A. gambiae*, in which two functional genes (AgSTAT-A and AgSTAT-B) have been characterized [13]. The AgSTAT-A gene is ancestral and is the putative ortholog of STAT genes from other insects. AgSTAT-B is an intronless gene that is evolving fast and appears to be the result of a retro-transposition event in which an AgSTAT-A cDNA was re-inserted back into the genome. Interestingly, AgSTAT-B regulates transcription of AgSTAT-A in adult stages and is the only STAT gene expressed in pupae [13].

In this work, three genes of the JAK-STAT pathway of *A. aquasalis*, the transcription factor STAT, its regulatory protein PIAS and NOS were cloned, sequenced and characterized. The domain organization of the PIAS protein is very similar to that of the *A. gambiae* and *A. aegypti* orthologs. The deduced *A. aquasalis* STAT, on the other hand, lacks some of the N-terminal conserved domains present in *A. gambiae*, *A. aegypti* and *Drosophila* STATs. It is probably the product of alternative splicing, as a similar cDNA (ΔN-STAT92) giving rise to a protein that lacks 113 aa at the N–terminus, has been characterized in *Drosophila* [32].

AqSTAT and AqPIAS mRNAs are expressed in all insect stages and both in males and females. The high expression in eggs and first instar larvae may be indicating that, as in *D. melanogaster* [33], [34], the JAK-STAT pathway in *A. aquasalis* may also participate in oogenesis and embryogenesis. The expression pattern of AqSTAT mRNA in adult stages is very similar to *A. gambiae* STAT-A [13], as in both anophelines males express higher STAT mRNA levels than sugar-fed females. In *A. gambiae*, AgSTAT-A expression remained unchanged 24 hours after infection with *P. berghei* [13]. In contrast, AqSTAT expression was activated transiently by *P. vivax* infection at 24 and 36 hpi. AqPIAS presented an mRNA expression pattern similar to AqSTAT and the induction of these two genes suggests that the JAK-STAT pathway is activated in response to *P. vivax* infection. The induction of PIAS protein expression corroborated the transcriptional results and provided direct evidence that the JAK-STAT pathway is also carefully regulated in *A. aquasalis*. Silencing AgSTAT-A in *A. gambiae* females infected with *P. berghei* reduced the number of early oocysts present two days post-infection, nevertheless enhancing the overall infection by increasing oocyst survival [13]. AqSTAT silencing also increased the number of oocysts, but its effect on very early stages of infection remains to be established. The peak transcriptional activation of the JAK-STAT pathway at 36 hpi was similar to what we observed for other immune genes such as serpins, bacterial responsive protein and fibrinogen [21], indicating that the immune system is activated at the time when *Plasmodium* parasites have invaded the midgut and come in contact with the mosquito haemolymph. The activation of the JAK-STAT pathway at this time of infection may be regulating hemocyte differentiation, as seen in *Drosophila* [35]. In the case of *A. aquasalis*, this could help killing parasites and controlling infection.

Immunocytochemistry revealed that *A. aquasalis* STAT and PIAS not only had concomitant expression but also localized in the same tissues. The expression of these proteins in sugar-fed males and females was mostly observed in the fat body, with males presenting stronger labeling than females. This corroborated the role of the fat body as the main immune organ of the insects. The detection of high levels of protein in males is in agreement with our previous results for other *A. aquasalis* immune genes such as fibrinogen, bacteria responsive protein and cecropin [21]. This seems to indicate that male mosquitoes are more prepared for eventual challenges, as opposed to what was observed in vertebrates and some invertebrate species, where females are more immunocompetent than males [36]. The expression of STAT and PIAS also presented differences between non-infected and infected insects. The non-infected insects were immunologically marked mainly in the fat body while the infected ones were marked in dispersed cells along all body and in the ingested blood. This pattern of expression of proteins from the JAK-STAT pathway demonstrated that *A. aquasalis* is producing a systemic immune response against *P. vivax*.

In vertebrates, STAT1 regulates NOS expression [37]. DNA sequences capable of binding to STAT and NF-κB have been described in the regulatory regions of the NOS gene in *A. stephensi* [38]. In *A. gambiae*, AgSTAT-A participates in the transcriptional activation of NOS in response to bacterial and plasmodial infections, NOS expression being activated by *P. berghei* 24 hpi [13]. In *A. aquasalis*, we observed high levels of NOS transcription at a later time (36 hpi) in response to *P. vivax*. Luckhart *et al.* [39], [40] detected an increase in *A. stephensi* midgut NOS mRNA at several times (6, 24, 48 and 72 h) after *P. berghei* infection. In *A. gambiae* infected with *P. falciparum* induction of NOS mRNA was also observed [41]. High expression of NOS protein was also seen in the cytoplasm of some midgut cells of *A. aquasalis* 24 hpi. These observations suggest that activation of the JAK-STAT pathway may be regulating NOS expression and that NO may be an important mediator of the antiplasmodial response. In some models of vector-parasite interaction as *A. stephensi-P. berghei*, insect midgut cells suffer damage after parasite invasion. Among these are protrusions toward the lumen, loss of microvilli, induction of NOS and production of NO, which is converted into nitrite and then into NO_2_, causing protein nitration that leads to cell death [42], [43]. This epithelial immune response is important to control parasite numbers and, in some cases, can be decisive for clearance of infection. Nevertheless, this mechanism is not universal, as induction of NOS and peroxidase activities were not observed in other vector-parasite combinations such as *A. aegypti–Plasmodium gallinaceum* and *A. stephensi–P. gallinaceum* [44]. The apparent inconsistency in the timing of appearance of NOS protein in the midgut and mRNA levels for this gene might be due to the expression of NOS mRNA only in the cells of the infected midgut injured by the parasite passage. Moreover, the expression of the mRNA in others organs of the insect can explain this discrepancies since the mRNA experiments were performed with whole mosquitoes and the protein expression only with the midgut.

Our results showed that the *A. aquasalis* JAK-STAT pathway is activated in response to *P. vivax* challenge. Furthermore, preventing activation of the JAK-STAT pathway by silencing the AqSTAT transcription factor increased the infection, as well as the number of *P. vivax* oocysts in *A. aquasalis* mosquitoes. These results confirm the role of the JAK-STAT in limiting *P. vivax* infection of *A. aquasalis*. Enhancing these responses by using a transgenic approach may be effective in preventing *P. vivax* malaria transmission to humans by *A. aquasalis* mosquitoes.

## Supporting Information

Figure S1 **Sequence of STAT obtained from PCR fragments produced using degenerate primers and RACE PCR.** Numbers on the left indicate nucleotide sequence length and on the right indicate amino acid sequence length; asterisk indicates the stop codon; aminoacids in italics represent the hydrophobic binding pocket; the aminoacids in bold format indicate the phosphotyrosine binding pocket; the underlined aminoacids represent the alpha domain; the dashed aminoacids represent the binding domain; uperlined aminoacids indicates the SH2 domain. The nucleotides in bold format indicate the poly(A) tail. AqSTAT sequence was deposited under accession HM851178.
(TIF)

Figure S2 **Sequence of PIAS obtained from PCR fragments produced using degenerate primer and RACE PCR.** Numbers on the left indicate nucleotide sequence length and on the right indicate amino acid sequence length and asterisk indicates the stop codon. The underlined aminoacids represent the SAP domain and the dashed the MIZ/SP-RING zinc finger domain. The nucleotides in bold format indicate the poly(A) tail. AqPIAS sequence was deposited under accession number HM851177.
(TIF)
