# Supplementary figures and images for: The JAK-STAT Pathway Controls Plasmodium vivax Load in Early Stages of Anopheles aquasalis Infection
Source: PLoS Negl Trop Dis. 2011 Nov 1;5(11):e1317. doi: 10.1371/journal.pntd.0001317 (PMC3206008; doi:10.1371/journal.pntd.0001317)

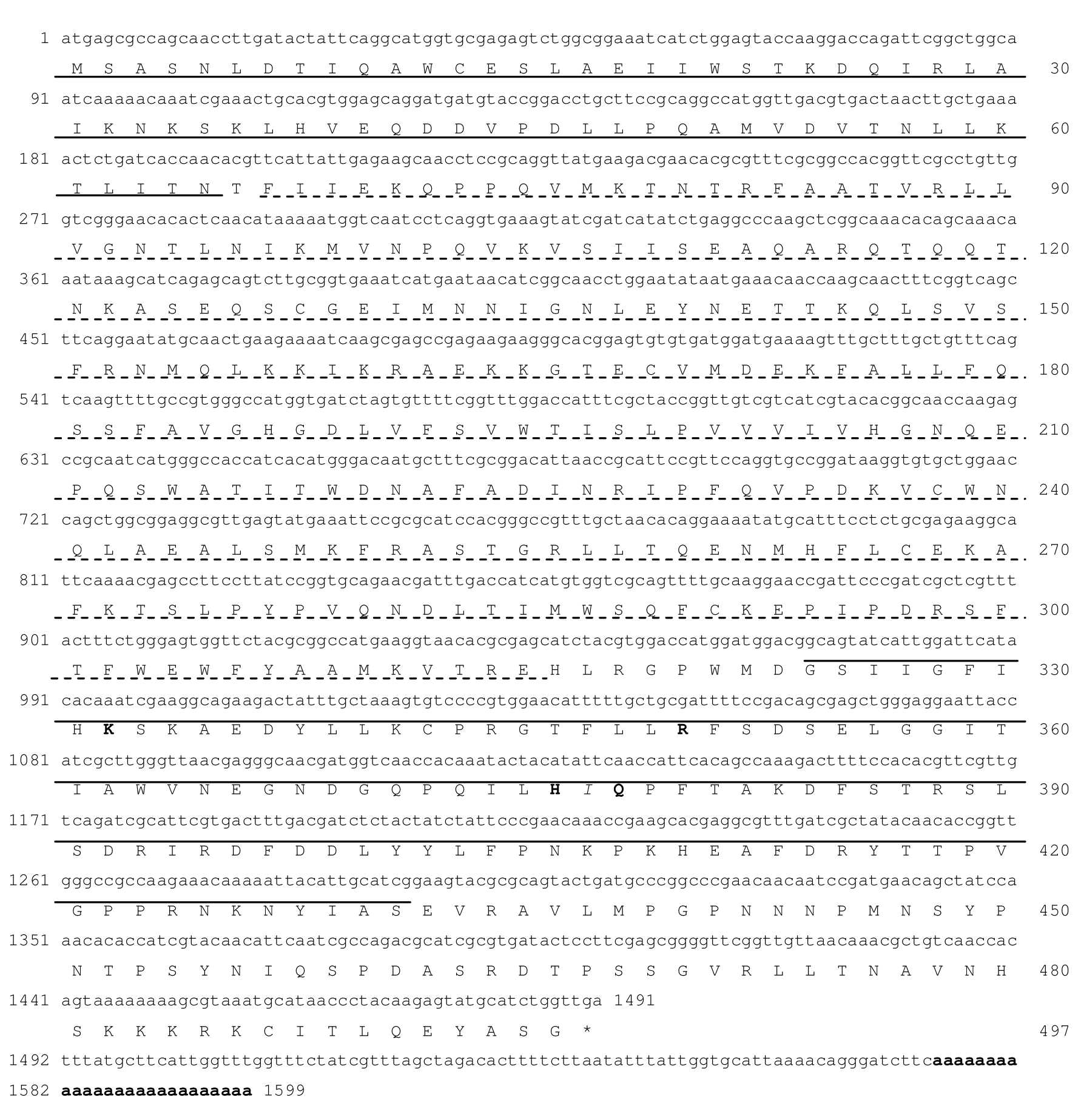

Supplement: Figure S1 — Sequence of STAT obtained from PCR fragments produced using degenerate primers and RACE PCR. Numbers on the left indicate nucleotide sequence length and on the right indicate amino acid sequence length; asterisk indicates the stop codon; aminoacids in italics represent the hydrophobic binding pocket; the aminoacids in bold format indicate the phosphotyrosine binding pocket; the underlined aminoacids represent the alpha domain; the dashed aminoacids represent the binding domain; uperlined aminoacids indicates the SH2 domain. The nucleotides in bold format indicate the poly(A) tail. AqSTAT sequence was deposited under accession HM851178. (TIF) [file pntd.0001317.s001.tif]

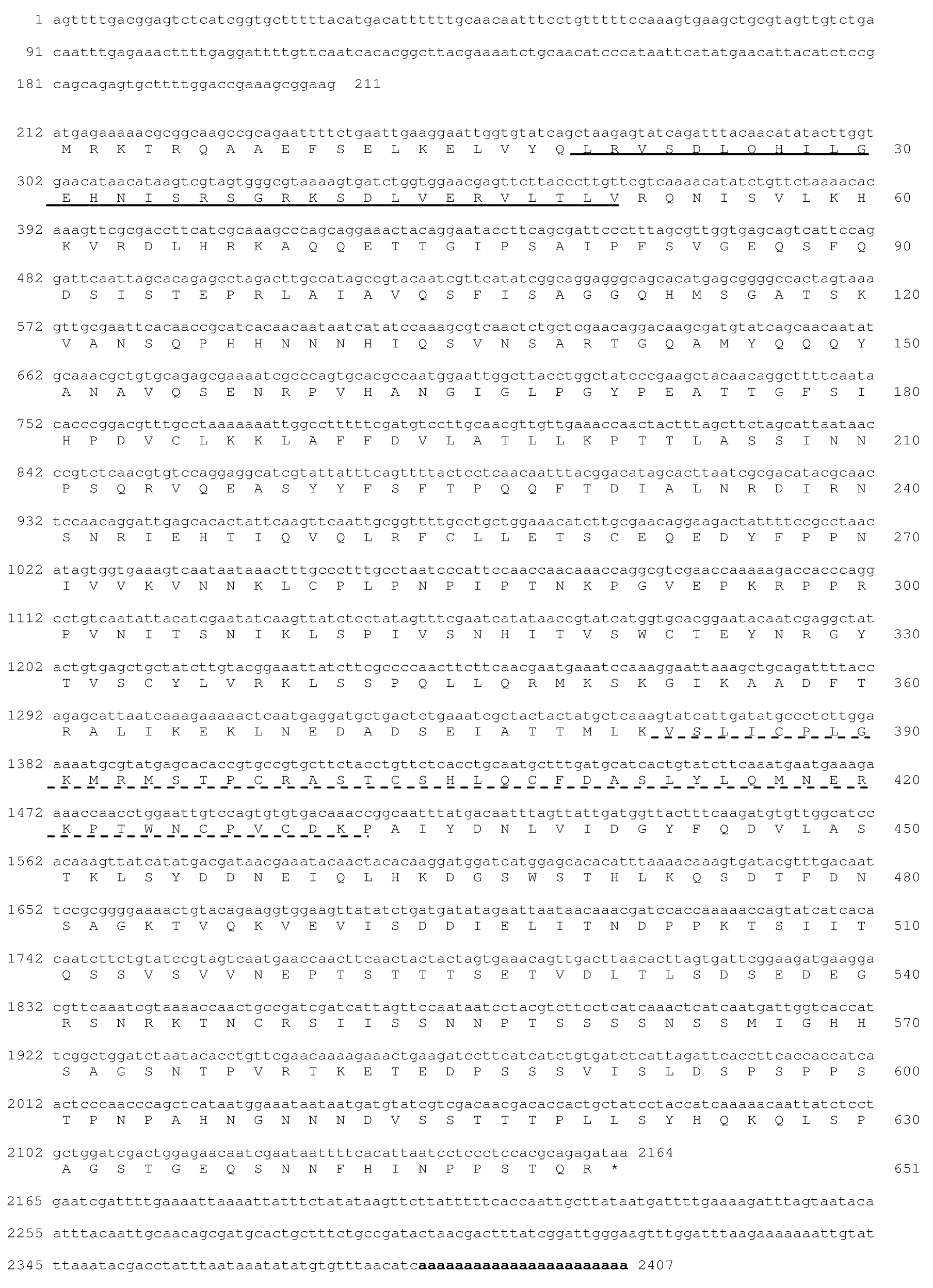

Supplement: Figure S2 — Sequence of PIAS obtained from PCR fragments produced using degenerate primer and RACE PCR. Numbers on the left indicate nucleotide sequence length and on the right indicate amino acid sequence length and asterisk indicates the stop codon. The underlined aminoacids represent the SAP domain and the dashed the MIZ/SP-RING zinc finger domain. The nucleotides in bold format indicate the poly(A) tail. AqPIAS sequence was deposited under accession number HM851177. (TIF) [file pntd.0001317.s002.tif]
